# Early sensitisation to food allergents in Lithuanian birth cohort

**DOI:** 10.1186/2045-7022-1-S1-O4

**Published:** 2011-08-12

**Authors:** Indre Butiene, Odilija Rudzeviciene, Ruta Dubakiene

**Affiliations:** 1 Vilnius University, Faculty of medicine, Vilnius, Lithuania; 2 Vilnius University, Clinic of Children's Diseases, Vilnius, Lithuania

## Background

The factors responsible for the induction of allergic disease at an early age have not been completely identified. Data about maternal factors during pregnancy and early sensitization to food allergens are limited. Aim: To determine the prevalence of food sensitization in children under 6 months of age and to determine relationship between maternal avoidance of allergenic foods, maternal disease, use of antibiotic, tobacco smoke during pregnancy and early sensitization to food allergens.

## Methods

The analysis was based on data of 1558 subjects from a EuroPrevall Lithuanian birth cohort study ( EU 6 FP project "EuroPrevall:). Children younger than 6 months of age and sensitized to food allergens and their controls were analyzed. Information was collected using parental questionnaires filled at the day of recruitment, 12 months questionnaire and physical examination form, results of SPT and sIgE analysis.

## Results

Early sensitization to food allergens was detected in 20 children under 6 months of age (1.3%, 1558). 15 (75%) symptomatic subjects were sensitized to milk, positive SPT was found in 5, elevated sIgE in 4, only immediate or repetitive symptoms were reported in 8 patients. 12 (60%) symptomatic subjects were sensitized to egg, positive SPT was found in 9, elevated sIgE in 7, only symptoms were reported in 1 patient. Sensitization to wheat was confirmed in 2 patients by SPT and reported symptoms, to peanut - in 1 subject by elevated sIgE. The food allergy was confirmed by positive DBPCFC in 4 infants - 2 for milk, 1 - egg, 1 - wheat. There were found no significant impact of maternal diseases (p = 0.34), use of antibiotics (p = 0.7), tobacco smoke (p = 0.25) during pregnancy on early sensitization to food allergens. Maternal avoidance of milk and egg products during pregnancy, as well as use in elevated amounts of the product was not related to early sensitization to milk and egg allergens (p = 0.38).

## Conclusions

The prevalence of food sensitization in children under 6 months of age was 1.3%. Maternal diet and disease, use of antibiotic, tobacco smoke during pregnancy had no significant impact on early sensitization to food allergens.

